# Statistical methodology for the evaluation of leukocyte data in wild reptile populations: A case study with the common wall lizard (*Podarcis muralis*)

**DOI:** 10.1371/journal.pone.0237992

**Published:** 2020-08-26

**Authors:** Roberto Sacchi, Marco Mangiacotti, Stefano Scali, Alan J. Coladonato, Silvia Pitoni, Mattia Falaschi, Marco A. L. Zuffi

**Affiliations:** 1 Dipartimento di Scienze della Terra e dell’Ambiente, Università degli Studi di Pavia, Pavia, Italy; 2 Museo di Storia Naturale di Milano, Milano, Italy; 3 Dipartimento di Scienze e Politiche Ambientali, Università degli Studi di Milano, Milano, Italy; 4 Museo di Storia Naturale, Università di Pisa, Calci, Italy; University of Minnesota, UNITED STATES

## Abstract

The leukocyte profile has the potential to be a reliable method to measure health conditions and stress in wild animals, but limitations occur because current knowledge on reference intervals is largely incomplete, especially because data come from studies on captive animals involving few individuals from single populations. Here we propose a general framework for achieving reliable leukocyte reference intervals, encompassing a set of internal and external factors, potentially affecting the leukogram. To do so, we present a systematic survey of the hematology of the common wall lizard, *Podarcis muralis*, involving 794 lizards from 54 populations over the whole geographic range of the species in Italy. Reference intervals for white blood cell (WBC) and leukocyte differential count were obtained by using linear mixed models in a Bayesian framework. The application of the procedure clearly showed that both internal (sex and size) and external (latitude and season) factors are a source of variation of leukocyte profile. Furthermore, the leukogram of common wall lizard has a strong variability among populations, which accounts for more than 50% of the whole variation. Consequently, some common assumptions used in studies on captive individuals are no longer supported in wild populations, namely, i) any group of individuals is a representative sample, ii) any population is representative of all others, iii) geographic clines do not occur over the species range, and iv) seasonal variation has limited effects. We encourage researchers aimed at the definition of leukocyte reference intervals for wild populations of reptiles to involve a large number of populations over a wide geographic range in *ad hoc* statistical models to disentangle local and geographic effects on leukocyte profile variation.

## Introduction

Characterizing the physiological responses of wild animals to stressors, including human-induced landscape changes, is an important question with deep implications for both animal conservation and ecology [1]. Indeed, stress is a key factor when dealing with animal welfare in both wild and captive contexts [1, 2], as well in determining how resource investment in immune-function shapes species life-history traits [3].

Leukocyte profile has the potential to be a reliable method to measure stress in vertebrates, as an alternative to hormone assay [1]. For example, neutrophils (heterophils in birds and reptiles) and lymphocytes respond in the opposite way to stress, so that their ratio is positively related to the circulating glucocorticoids and the magnitude of the stressor [4]. The increase of the total number of leukocytes (leukocytosis) has been occasionally used as a measure of stress [reviewed in 1] as well as glucocorticoid-induced stress has been shown to reduce the eosinophil number, even if only in humans and other mammals [reviewed in 1]. Moreover, the increase in the number of eosinophils (eosinophilia) has also been used as a measure of stress, although with contrasting results [1]. Since the lower end of eosinophil reference intervals frequently include zero, and eosinophils typically represent a minor part of the leukogram in many species, changes to eosinophils are typically not used to assess for stress in human or veterinary medicine. Compared to hormone assay, the enumeration of white blood cells from blood smears is cheaper and makes the assessment of the baseline much easier to do since the initial response of leukocytes begins within hours (or in a few days) after the stress. The downsides are essentially two: the interpretation of leukocyte counts is difficult, and the leukocyte profile does not necessarily supply information about the ability of individuals to mount an immune response [1].

The potential of the leukocyte profile as a tool in ecological and conservational research can be fully realized only after robust information on reference values for “normal” individuals is achieved. Reference values (RV) describe the variation in blood analyte concentrations in well characterized groups of healthy individuals, while Reference intervals (RI) represent estimated distributions of RV from healthy populations of comparable individuals [5]. Population-based RI have become one of the most common tools used in clinical decision-making processes [5]. To be reliable for wild individuals, RI should be assessed by adopting general guidelines for the selection of reference individuals, which must embrace the variability among populations of the focal species [5]. Without this information, disclosing if the cell count of a given individual is high or low compared to the leukocyte profile of healthy individuals is, in fact, impossible. Unfortunately, many of the RI proposed so far have been obtained with captive animals or, when done in the wild, by a survey of only a few (even only one) populations (Table 1). Indeed, the approach used to date in ecological and conservational research has been borrowed from veterinary research and has involved samples generally around 50–70 units, from a single locality, without replication (Table 1). Such a kind of approach can provide reliable data when dealing with pets or farm animals, which live in strictly controlled conditions that sensibly reduce all those sources of variation in leukocyte profile proper to natural conditions, such as habitat heterogeneity, climatic variability, food distribution, and competition with other species, just to name a few. The effect of these external factors on leukocyte profile in wild populations, however, cannot be disregarded, as they can be one of the main causes of variation among populations, all over the range of the species [6, 7].

**Table 1:** Methodological approaches of studies reporting reference values for the leukocyte differential count in reptiles.

Species	N ind.	Condition	N populations	Internal factors	External factors	Reference
*Anguis fragilis*	47	Wild	Random collection	Sex	Season	[8]
*Emys orbicularis*	34	Semi-captive	--	Sex	Season	[9]
*Podarcis muralis*	43	Wild	1	Sex	Season	[9]
*Natrix maura*	121	Wild	1	Sex	Season	[9]
24 species	< 3	Wild/Captive	1	Sex	--	[10]
*Pogona vitticeps*	21	Captive	--	--	--	[11]
*Gopherus agassizii*	98	Wild	3	Sex	Season, site	[12]
*Crocodylus palustris*	54	Captive	--	Sex, age	--	[13]
*Gopherus agassizii*	36	Wild	3	Sex	Season, year, site	[6]
*Ophiophagus hannah*	28	Wild/Caprive	1	Sex	--	[14]
*Bothrops leucurus*	29	Wild	1	Sex	--	[15]
*Mauremys leporosa*	114	Wild	1	Sex	--	[16]
*Tupinambis merianae*	100	Semi-captive	1	Sex, age	Season	[17]
*Heloderma horridum*	16	Wild	1	--	--	[18]
*Caiman latirostris*	24	Captive	--	--	--	[19]
12 lacertid lizards	7–81	Wild	1	Sex	--	[20]
4 agamid lizards	10–20	Wild	1	Sex	--	[21]
*Pogona vitticeps*	100	Captive	--	Sex	Season	[22]
*Hieremys annandalii*	40	Wild/semi-captive	1	Sex	--	[23]
*Thamnophis gigas*	49	Wild	4	--	Site	[7]
*Thamnophis sirtalis*	39	Wild	4	--	Site	[7]
*Vipera ammodytes*	31	Wild	1	Sex	--	[24]
*Mauremys mutica*	53	Captive	--	Sex	Season	[25]
*Oxyrhopus guibei*	26	Wild	Random collection	Sex	--	[26]
*Xenodon neuwiedii*	19	Wild	Random collection	Sex	--	[26]
*Testudo hermanni*	34	Captive	--	Sex	Season	[27]
*Naja naja*	59	Wild	1	Sex	--	[28]
*Tiliqua rugosa*	33	Captive	--	--	--	[29]
*Thamnophis sirtalis*	86	Wild	12	Sex	Year, site	[30]
*Emydoidea blandingii*	386	Wild	2	--	Site	[31]
*Pituophis ruthveni*	11	Wild	1	Age	--	[32]
*Podarcis muralis*	794	Wild	54	Sex, age	Season, latitude, site	Present study

Although differences between captive and free-ranging individuals have been documented in reptiles [14, 16, 24, 33], the lack of RI for wild populations is particularly severe for this clade [34]. Many authors have described the morphology of reptiles’ blood cells, particularly for species of commercial interest, such tortoises, crocodiles, and iguanas [10, 33, 35]. Several attempts to determine RI for leukocyte differential counts have also been performed, but mostly involved captive individuals or one or a few populations (Table 1). The first attempt dates back to the work by Duguy [8, 9], whereas the first review on RI for leukocytes in reptiles is by Frye [10], who reported the leukocyte differential count for 24 species of reptiles (including four tortoises, fourteen lizards, five snakes, and one crocodile). However, in all but one case, no more than three individuals for species were considered. Since then, several studies have investigated reptilian hematology in wild individuals, but always with the limitation of using a few individuals collected from one/few populations (Table 1). During the last decades a large amount of data has also been collected in parallel for veterinary purposes, but even in this case, the large majority of studies involved few individuals and did not account for possible seasonal variation neither did cover a significant portion of the geographic range of the species (Table 1). To date, only a very restricted number of studies have focused on reptiles hematology in wild populations over large geographic ranges [6, 26, 30]. Dickinson et al. [6] supplied RI (with seasonal and annual variation) for 36 radio-tagged *Gopherus agassizii* sampled in three localities of the Sonoran desert, Ozzetti, et al. [26] supplied leukocyte baseline for *Oxyrhopus guibei* and *Xenodon neuwiedii* in Brazil collecting individuals opportunistically, leading to sample *de facto* only one individual for each locality, whereas Gangloff et al. [30] supplied a baseline for the heterophils to lymphocytes ratio on a sample of 86 *Thamnophis sirtalis* collected in 12 populations widespread over the distribution range in North America. Given the lack of reliable RI, there is the need to develop standardized techniques [5] to collect data from wild populations, to be able to properly use leukocyte profiles in ecological and conservation studies of reptiles.

A further issue regarding the reliability of the RI concerns the statistical treatment of data used to define them [5]. Most studies compare groups of individuals (mostly males and females) by simple one-way analyses (i.e., t-test or one-way ANOVA), and established intervals reporting ranges as well as standard deviations/errors of the observed data. However, such a kind of intervals only describes the probability of obtaining the same result if the measure is replicated but does not allow any inference on the possibility of classifying new observations. No previous studies have explicitly implemented the effect of the breeding season in the analyses, nor the effect of the geographic variability.

We believe that reliable RI for ecological and conservational purposes can only be achieved through the application of a general, well-defined protocol encompassing the patterns of variation of leukocytes, due to both internal (age and sex) and external factors (season and latitude) potentially affecting leukocyte parameters. Furthermore, we also think that RI for leukocytes should be estimated through an appropriate statistical model and not directly from the raw data. Only in this way it is possible to rule out the effects of the external and internal perturbations to leukocyte parameters and estimate the baseline of RV. In summary, to overcome the above issues, the best solution would be to use: 1) large sample within the population including individual from both sexes and of different age classes; 2) repeated sampling within the population to account for seasonal variation of leukocytes’ relative abundances; 3) sampling data from different populations settled all over a large portion (possibly the whole range) of the distribution of the species; 4) appropriate statistical models that allow to decompose the variance among individuals according to the internal and external factors involved, and to assess the among-populations variation.

The common wall lizard has been extensively studied in light of hematology and immune function [e.g., 20, 36–40]. It is a small lacertid lizard (snout-vent length, SVL, 45–75 mm) widespread in Southern and Central Europe, which produces on average two clutches per year [41]. The species is sexually dimorphic [42] and the breeding season starts in late February and ends in July [41]. Body temperature during activity is near 33 °C, being slightly higher (33–36 °C) in warmer regions (e.g., Central Italy) and lower (32 °C) in mountain areas [43–45]. Morphology of blood cells has been repeatedly studied [20, 36, 37, 39] as well the immune response has been previously investigated concerning polymorphic ventral coloration [40, 46], immuno-competence handicap hypothesis [38], or temperature [40, 47]. Despite this, RI for the leukocyte profile in wild populations still lack, though some pivotal studies have been performed in single, isolated populations [39].

In this paper, we applied the suggested framework to the analysis of the common wall lizard (*Podarcis muralis*) leukocyte profile, with a double aim in mind: i) quantifying the bias we could introduce in the RI estimates by ignoring (i.e., not measuring) the among-population, seasonal, and geographical variability; ii) measure the performance of our procedure when dealing with those sources of variation, and its ability to eliminate or control for the effects of outliers.

## Materials and methods

**Fig 1:**
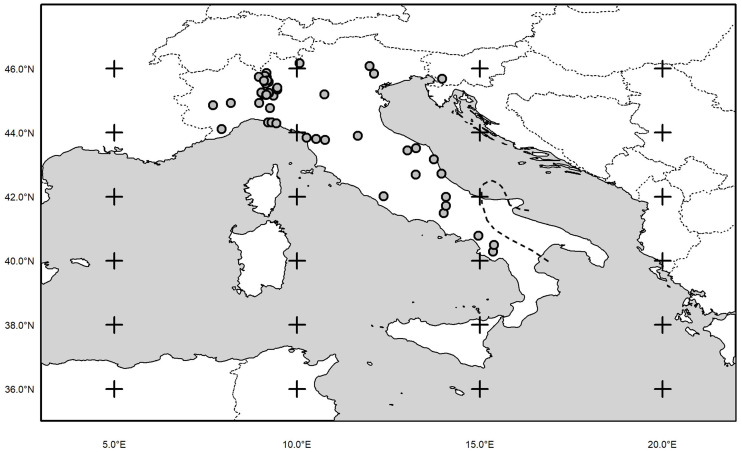
Distribution map of the 54 populations of the common wall lizard (*Podarcis muralis*) sample all over Italy. The black dashed line delimit southern range of the species in continental Italy (maps have been downloaded at the Natural Earth Data (http://www.naturalearthdata.com).

Adult common wall lizards were collected by noosing during March-September between 2008 and 2017 in 54 sites within the whole species distribution in Italy (Fig 1; Table 2). Only lizards in apparently healthy and in good condition (external examination) were selected for this study and were handheld only for the time needed for measuring and blood collecting. The Ministry of Education, University and Research (MIUR) provided all the authorizations for the study (2008: Aut. Prot. PNM- 0003606; 2009–2011: Aut. Prot. PNM-0020292; 2012–2013: Aut. Prot. PNM-0009344; 2014–2015: Aut. Prot. PNM-0011379; 2016–2018: Aut. Prot. PNM-0002154). Overall, we captured 794 individuals (498 males and 296 females), and on average (± SE) 10 ± 2 males (range: 2–77) and 9 ± 1 females (range: 2–38) for each population. After capture, the snout-vent length (SVL) of each individual was measured with a digital caliper. Males and females sized on average (± SE) 63.6 ± 0.2 mm (range: 47.1–79.4 mm) and 59.4 ± 0.3 mm (range: 43.5–76.2 mm) respectively. Common wall lizards reach sexual maturity when larger than 50 mm if females [41], and 59 mm if males [48].

**Table 2:** Timing of captures according to location of sites.

	N. sites	Mean	Range
Northern Italy	30	30^th^ May	13^th^ February–6^th^ October
Central Italy	14	2^nd^ June	2^nd^ April–15^th^ August
Southern Italy	10	28^th^ May	27^th^ April–5^th^ September

Blood samples (15–20 μl) were collected in the field, from the postorbital plexus, using heparinized capillary tubes, after holding the head of the lizard firmly in one hand [49]. Soon after blood sampling individuals were released healthy in the capture site. Air-dried smears were stained with May–Grünwald/Giemsa stain and scanned using a light microscope (OPTIKA) at 60× following standard routines. In each microscope field, leukocytes were counted and classified as lymphocytes, monocytes, eosinophils, heterophils, or basophils. In each smear, we explored no less than 50 fields for no less than 150 leukocytes (i.e., if after 50 fields the count of leukocytes did not exceed 150, new fields were added until this value was reached). This ensured us to be able to detect leukocytes until a frequency of 0.7%. Total white blood cell counts (WBC) were estimated from May–Grünwald/Giemsa stained blood films by multiplying the mean number of leukocytes per microscopic field by the objective power squared [50].

### Statistical analyses

The WBC were log-transformed to achieve normality. Leukocyte differential count is inherently a composition (with dimension D = 5, [51]), and cannot be used in linear model as it stands. Therefore, we used the isometric log-ratio transformation (ilr) to map the leukocyte differential count of each individual in a D-1 Euclidean space, thus generating a set of four independent variables [51]. To account for seasonal variation of leukocyte variables, we used multiple-component cosinor models, which were first developed by Halberg et al. [52] to model physiological processes that have a circadian rhythm. Those models use linear combinations of cosine functions to assess the amplitude (A) and phase (φ) of physiological rhythms around the average value (i.e. the midline estimating statistic of rhythm, MESOR) over the period (τ). We used a single-component cosinor model, i.e., Y(time) = MESOR + Acos(2π(time)/τ + *ϕ*) + e(time) including time expressed as Julian date (1 = January 1^st^) and τ = 365 to account for effect of circannual rhythms around the baseline of each leukocyte variable. This model was incorporated into a random intercept Linear Mixed-Model (LMM) to assess the combined effects of circannual rhythm, biotic, and abiotic factors on lizards’ leukocyte profile. Cosinor, sex, body size (standardized SVL), and latitude (standardized UTM coordinate) entered the model as fixed effects. We also added the cosinor × sex, cosinor × body size, and cosinor × latitude interactions to account for possible sex, size, and geographical specific patterns in circannual rhythms on leukocyte variables. The population entered the model as a random intercept to account for unexplained variation at the population level (σ^2^_pop_) when controlling for the explanatory variables. An independent model was carried out for each hematological variable. Models were fit in a Bayesian analytical framework available in the package R2jags [53] in R 4.3.3 [54], which uses the samplers implemented in JAGS 4.3.0. Uninformative normal priors (μ = 0 and σ = 0.001) were used for model’s coefficients, and gamma priors (a = 0.001 and b = 0.001 corresponding to μ = 1 and σ = 1,000) were used for both the error (σ) and the random intercept (σ_pop_). Three chains were run using randomly selected initial values for each parameter within a reasonable interval, and conventional convergence criteria were checked. The number of iterations was selected for each run to obtain at least 10,000 valid values for each chain after convergence and thinning. The posterior distributions were back-transformed to obtain the posterior distributions of the variables in their original scales. According to ASVCP guidelines [5], baselines and RI were extracted from the posterior distributions of the values predicted by the models, as the mode and 95% prediction intervals.

## Results

### WBC

**Fig 2:**
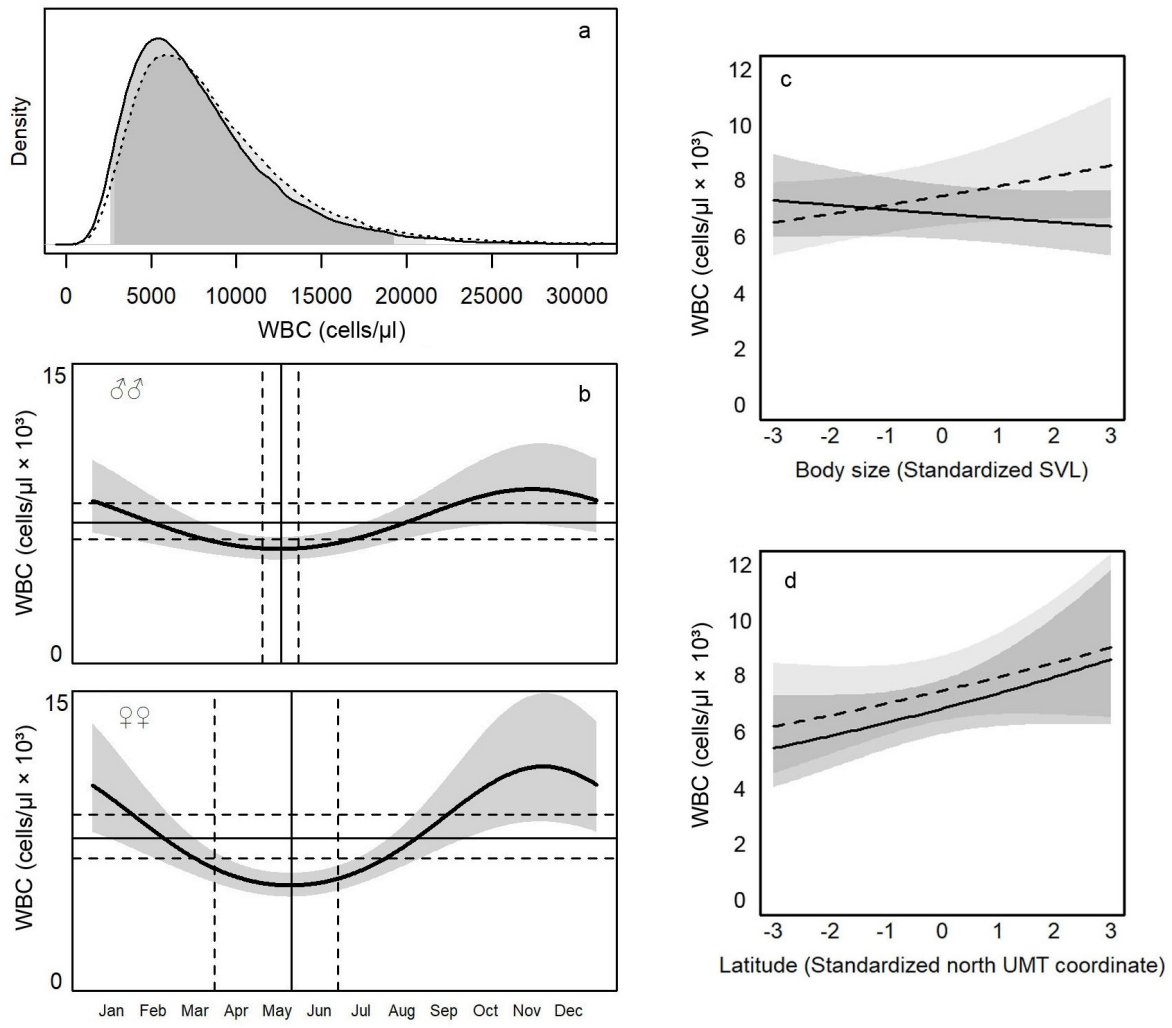
White blood cells (WBC) counts in common wall lizards (*Podarcis muralis*) in Italy as estimated by Linear Mixed Models (LMMs); a) posterior distributions with 95% credible intervals (see Methods for details) in males (solid line and dark gray) and females (dotted line and light gray); b) seasonal variation of WBC in males (upper panel) and females (lower panel). Solid lines and gray areas represent respectively medians and 95% credible intervals as predicted by the model; horizontal lines represent the midline estimating statistic of rhythm (MESOR, solid) and the corresponding 95% credible intervals (dotted); vertical lines represent the phases (solid) and the corresponding 95% credible intervals (dotted); c) effect of body size (standardized SVL) in males (solid line) and females (dotted line). Gray areas represent 95% credible intervals; d) effect of latitude (standardized UTM coordinate) in males (solid line) and females (dotted line). Gray areas represent 95% credible intervals.

The posterior distribution for WBC was skewed, with a longer upper tail (Fig 2a), suggesting that high values, even three-time greater than the mode of the distribution, of these two parameters could occur in wild individuals, albeit with low probability. Consequently, the RI for WBC as derived by the mode and the 95% prediction intervals of posterior distributions are sensibly asymmetric compared to the mean (Table 3). Accordingly, the probability of finding individuals with WBC exceeding the mode was 0.70, but it falls to 0.21 for individuals with WBC twice as high as the mode, until 0.05 for individuals with WBC three times the mode (despite two and three times the mode were both within the 95% prediction intervals).

**Table 3:** Reference values (mode and 95% prediction interval of the posterior distribution of values predicted by the models) for blood cells of the common wall lizards computed over 54 Italian populations, after controlling for the effect of season, latitude and body size.

Blood values	Males (n = 498)	Females (n = 296)
WBC (cells/μl)	5360 (2523–19289)	5829 (2757–21280)
Heterophils (%)	2.5 (0.9–21.9)	3.8 (1.4–30.7)
Eosinophils (%)	2.7 (1.0–23.2)	2.9 (1.0–24.1)
Basophils (%)	2.0 (0.7–21.8)	1.7 (0.6–18.0)
Lymphocytes (%)	83.4 (43.5–93.5)	78.2 (36.3–91.9)
Monocytes (%)	1.1 (0.3–26.0)	1.6 (0.4–34.1)

Gender differences emerged in both WBC, as highlighted by the posterior distributions of the differences between male and female MESORs. The concentration of leukocytes was higher in females than in males (β_♀-♂_ = 652 ± 546 cells/μl, P_β>0_ = 0.89<).

The models supported the occurrence of seasonal patterns of variation above and below the MESORs in WBC (Fig 2b). Indeed, the amplitude of WBC exceeded RI in both males (β_A-MESOR_ = 820 ± 679 cells/μl; P_β>0_ = 0.90, Fig 2b) and females (β_A-MESOR_ = 2792 ± 1248 cells/μl; P_β>0_ = 0.99, Fig 2b). Males reached the minimum of WBC on May 18^th^ (95% CI: May, 4^th^–May, 30^th^), and females a week later on May 25^th^ (March, 31^th^–June, 28^th^). The phase of oscillation did not differ between sexes (β_♀-♂_ = 5 ± 25, P_β<0_ = 0.50), whereas the amplitude of female was wider than that of males (β_♀-♂_ = 2213 ± 1272 cells/μl, P_β>0_ = 0.99).

WBC sensibly changed with body size (Fig 2c): with increasing body size, WBC increased in females (β = 361 ± 230 cells/μl, P_β>0_ = 0.95), but not in males (β = -157 ± 153, P_β<0_ = 0.86), showing a sex-based difference (β_♀-♂_ = 518 ± 259, P_β>0_ = 0.98).

Finally, we found a clear latitudinal cline in WBC (Fig 2d), which increased from South to North in both sexes (β_♂_ = 569 ± 348 cells/μl, P_β>0_ = 0.96; β_♀_ = 504 ± 382 cells/μl, P_β>0_ = 0.91). The slope did not differ between sexes (β_♀-♂_ = 64 ± 237 cells/μl, P_β>0_ = 0.61).

### Leukocyte differential count

**Fig 3:**
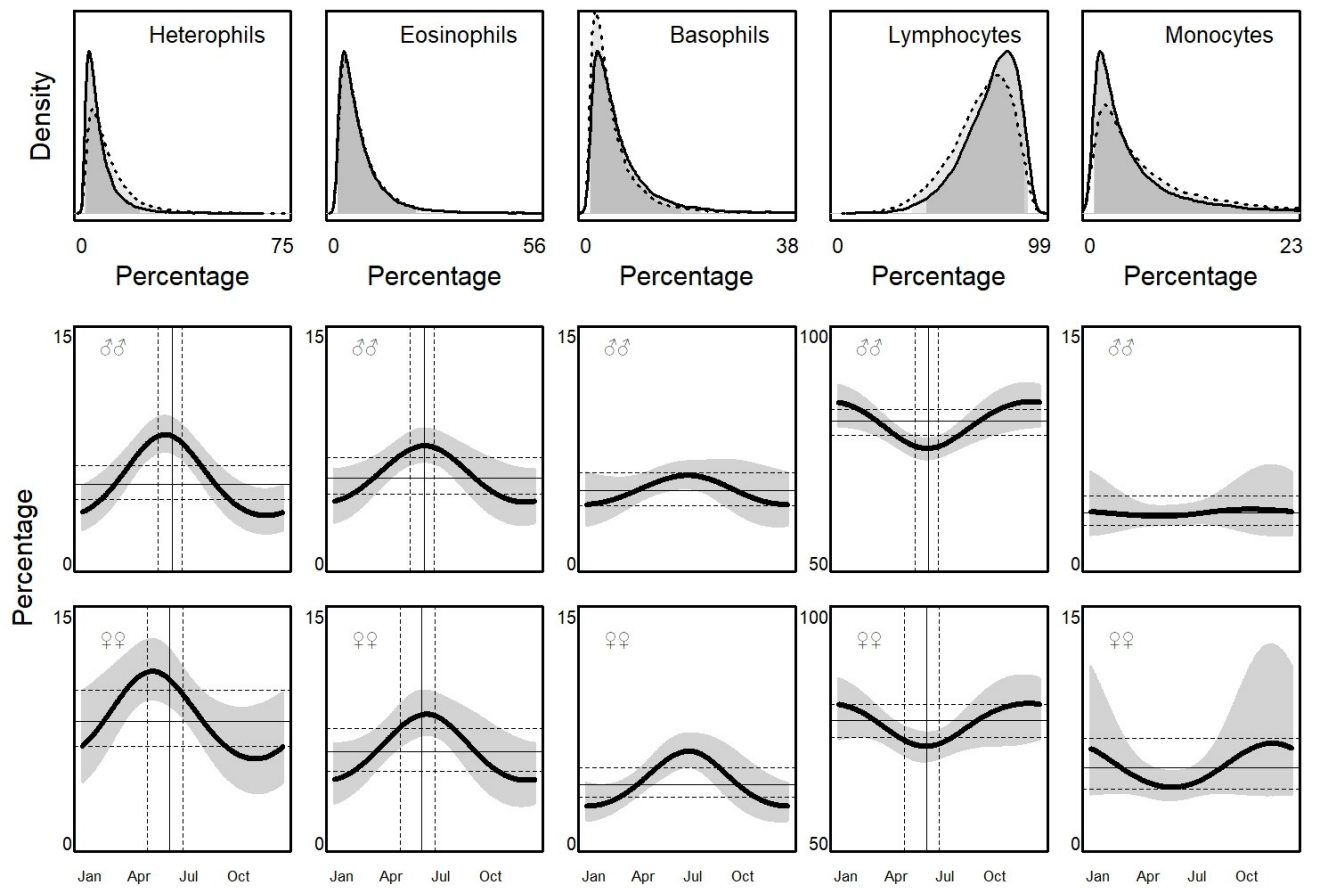
Leukocyte differential count as estimated by Linear Mixed Models (LMMs) and seasonal variation in males and females common wall lizards (*Podarcis muralis*) in Italy. Symbols as in Fig 2.

As for WBC, the posterior distributions of leukocytes’ relative abundances were sensibly skewed too, with a longer and shorter tail (Fig 3). Consequently, the 95% prediction intervals (i.e., the RI) were highly asymmetric (Table 3).

Differences between sexes in favour of females were found for the proportions of heterophils (β_♀-♂_ = 2.9% ± 1.0, P_β>0_ = 0.99), and monocytes (β_♀-♂_ = 1.7% ± 0.9, P_β>0_ = 0.98). At the opposite, males had more lymphocytes (β_♀-♂_ = -4.1% ± 2.0, P_β<0_ = 0.98), and basophils (β_♀-♂_ = -0.9% ± 0.6, P_β<0_ = 0.92) than females.

The models supported the occurrence of seasonal patterns of variation above and below the MESORs also for leukocyte differential count (Fig 3). The amplitude exceeded the RI of baseline (i.e., MESOR) in heterophils (♂♂: β_A-MESOR_ = 0.9% ± 0.2, P_β>0_ = 0.99; ♀♀: β_A-MESOR_ = 1.7% ± 0.5, P_β>0_ = 0.99), eosinophils (♂♂: β_A-MESOR_ = 0.2% ± 0.4, P_β>0_ = 0.72; ♀♀: β_A-MESOR_ = 0.1% ± 0.5, P_β>0_ = 0.68) and lymphocytes (♂♂: β_A-MESOR_ = 1.5% ± 1.2, P_β>0_ = 0.89; ♀♀: β_A-MESOR_ = 0.7% ± 1.9, P_β>0_ = 0.66). For basophils and monocytes, the chance for the amplitude to exceed the upper limit of RI was less than 0.43. The amplitudes were wider in females than in females for heterophils (β_♀-♂_ = 1.5% ± 0.5, P_β>0_ = 0.99) and eosinophils (β_♀-♂_ = 0.2% ± 0.6, P_β>0_ = 0.62), but were similar for lymphocytes (β_♀-♂_ = 0% ± 2.1%, P_β>0_ = 0.52). Heterophils and eosinophils increased during the breeding season, whereas the lymphocytes followed an opposite pattern (Fig 3). The peaks were similar between sexes (β_♀-♂_ = 5 ± 18, P_β>0_ = 0.45), and were on June 13^th^ (95% CI: May, 19^th^–July, 1^st^) in males, and on June, 9^th^ (95% CI: April, 30^th^–July, 3^rd^) in females.

**Fig 4:**
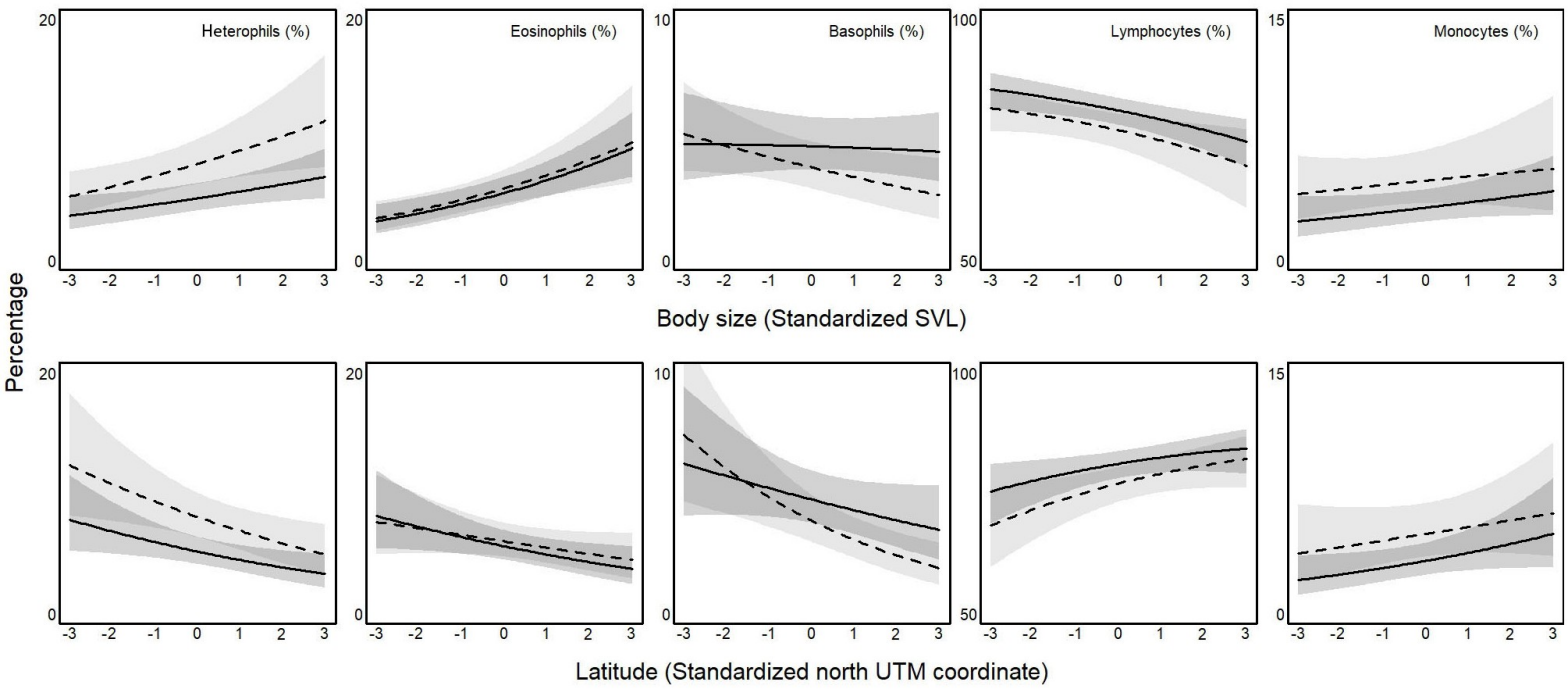
Effect of latitude (standardized UTM coordinate) on leukocyte differential count in males (solid line) and females (dotted line) common wall lizards (*Podarcis muralis*) in Italy. Gray areas are for 95% credible interval.

Leukocyte differential count changed according to body size, with similar patterns in males and females (Fig 4): values raised in larger individuals for heterophils (β_♂_ = 0.6% ± 0.2, P_β>0_ = 0.99; β_♀_ = 1.1% ± 0.5, P_β<0_ = 0.99), eosinophils (β_♂_ = 1.1% ± 0.3, P_β>0_ = 0.99; β_♀_ = 1.1% ± 0.4, P_β>0_ = 0.99), and monocytes (β_♂_ = 0.3% ± 0.2, P_β>0_ = 0.94; β_♀_ = 0.3% ± 0.4, P_β>0_ = 0.76), whereas they did the opposite for lymphocytes (β_♂_ = -1.9% ± 0.7, P_β<0_ = 0.99; β_♀_ = -2.1% ± 0.9, P_β<0_ = 0.99). Furthermore, basophils decreased with increasing body size in females (β = -0.4% ± 0.2, P_β<0_ = 0.98), but not in males (β = -0.1% ± 0.2, P_β<0_ = 0.64). Consequently, there was no difference between sexes in any leukocyte class (β_♀-♂_ < 0.2% ± 0.3, P_β>0_ <0.69), but heterophils (β_♀-♂_ = 0.6% ± 0.5, P_β>0_ = 0.86) and basophils (β_♀-♂_ = -0.3% ± 0.3, P_β>0_ = 0.91).

The last result concerned the effect of latitude, which was large as on most leukocyte classes (Fig 4). Leukocyte differential count changed with latitude in both sexes: going northwards, the proportion of lymphocytes (β_♂_ = 1.3% ± 0.7, P_β>0_ = 0.97; β_♀_ = 2.1% ± 0.9, P_β>0_ = 0.99) and monocytes (β_♂_ = 1.3% ± 0.7, P_β>0_ = 0.97; β_♀_ = 2.1% ± 0.9, P_β>0_ = 0.99) raised with a concomitant decline of the proportion of heterophils (β_♂_ = -0.7% ± 0.3, P_β<0_ = 0.99; β_♀_ = -1.2% ± 0.4, P_β<0_ = 0.99), eosinophils (β_♂_ = -0.7% ± 0.3, P_β<0_ = 0.98; β_♀_ = -0.5% ± 0.3, P_β<0_ = 0.94), and basophils (β_♂_ = -0.4% ± 0.3, P_β<0_ = 0.95; β_♀_ = -0.8% ± 0.2, P_β<0_ = 0.99). Consequently, the latitudinal effects (i.e., slopes) on males and females were similar in eosinophils (β_♀-♂_ = -0.2% ± 0.3, P_β<0_ = 0.31), and monocytes (β_♀-♂_ = 0.1% ± 0.4, P_β>0_ = 0.59), but differed between sexes in heterophils (β_♀-♂_ = 0.5% ± 0.4, P_β>0_ = 0.88), basophils (β_♀-♂_ = 0.4% ± 0.3, P_β>0_ = 0.91) and lymphocytes (β_♀-♂_ = -0.7% ± 0.9, P_β<0_ = 0.19).

### Among populations variation

We found a relevant among-populations variability (σ^2^_pop_) on all hematological variables, which accounted for a consistent proportion of the unexplained variation after we controlled for the explanatory variables. For WBC, it accounted for 57% of the total variation, whereas σ^2^_pop_ accounted for between 39% (lymphocytes) and 98% (eosinophils) of the total unexplained variation. The effect of population was negligible for monocytes (σ^2^_pop_ = 3%) and basophils (σ^2^_pop_ = 7%).

## Discussion

In ecological and conservation research, leukocyte profile has the potential to be a reliable method to measure stress and health condition in animals [1], as long as the reference values for wild and healthy individuals are fully available. The methodological approach used to assess.

RI is of primary importance, since the reliability of these values depends precisely on how they were obtained [5], and, as a cascade effect, also the validity of all decisions that are taken from these intervals. To date, there is no single and shared protocol to obtain reliable RI for wild animals (but see [5] for veterinary species), as evidenced by the outcome of our survey on previous studies on reptiles (Table 1). What is crucial, is that no one has yet made any attempt to test the reliability of those procedures, or to assess how important the effects of the variability among individuals and populations of the same species can be. In this study, we proposed a general method for defining RI for leukograms having a dual advantage: i) encompassing a set of internal (linked to individuals) and external (linked to the populations) factors, potentially affecting leukogram’s RI, and ii) assessing how much noise each of these factors causes to the RIs. These two goals can be achieved thanks to the use of linear mixed models for the definition of RIs instead of simple means and standard deviations on raw data as in previous studies. By using linear mixed models, baselines for RVs are obtained through the model intercept, while the effects, if any, of the external factors can be estimated through the model coefficients. In addition, the effect of the variability among populations can be assessed by inserting the population as a random effect into the model. Concerning previous approaches, the novelty of computing baselines and RIs through linear modeling is that upper and lower limits of the RI are estimated after removing the effects of both internal/external factors affecting leukocyte concentration and among-population variation over the distribution of the species. Furthermore, if models are computed under a Bayesian analytical framework, the RIs (i.e., 95% prediction intervals) represent the actual probability distribution of leukocytes of any individual in any population within the range of the species. In other words, intervals derived from the 95% prediction intervals gave us the range within which a new individual was predicted to fall with an accuracy of 95%. These ranges can be regarded as the natural variability of leukocyte parameters of the species and any deviation from it can be reasonably regarded as an indicator of possible diseases including inflammatory responses, stress, toxins, and leukemia.

The application of the procedure to wild populations of the common wall lizard data confirmed our hypothesis that both internal and external factors are a source of variation of the leukocyte profile. The direct implication is that some of the assumptions implicit in the studies in captive animals are no longer supported in studies involving wild populations, namely, i) any group of individuals is a representative sample, ii) any population is representative of all others, iii) lack of geographic clines over the species range, and that iv) seasonal variation has limited effects.

The assumption common to all previous studies that “all individuals involved were healthy” (i.e., the values obtained could be regarded as baselines) and, therefore, representative of the species baseline, was violated as demonstrated by our analyses. Indeed, the probability of including non-healthy lizards into the sample was far from being negligible, as, for example, 3.4% of males and 1.6% of females included in the sample exceeded the upper limit of the RI (leukocytosis). These are not trivial deviations, but rather they can be a serious problem when RIs are estimated as means of raw data and intervals are derived using the minimum and maximum of observed values [6, 12]. Indeed, by using minimum and maximum of observed values, all sampled individuals showing extreme values are regarded as healthy, when they might actually suffering from some disease (false negative). Our results show that such an approach may supply not accurate reference and overestimate the range of variability of leukocytes in healthy individuals, leading to type II errors (i.e., false negative) when screening individuals for disease occurrence. We further point out that also the statement that “all individuals were in healthy condition given that none was showing any pathological condition”, which is often used to improve data reliability, is not fully supported, given that all lizards exceeding RI did not show any morphological or behavioral anomaly. Accordingly, the ASVCP guidelines for the determination of RI point out the need to include procedures for outlier detection [5], and our statistical methodology represents a reliable approach to deal with outliers and their effect on RI estimations.

Our analyses show that also the assumption that “any population can be regarded as representative of the whole species” is not reasonable for the common wall lizard. Indeed, we found a huge variability among populations in most RVs, and variation among populations explained much more than 50% of the whole variation in the leukocyte profile. Since the population was modeled as a random effect, variability at the population level was not related to sex, age, latitude, and season, and should, therefore, be regarded as a property intrinsic to each population. We should not be surprised by that. Rather, it is exactly what we should observe if the leukocyte profile records the stress and health condition of individuals. Indeed, much of the variation in leukocytes is expected to depend on the site-specific conditions that individuals are experiencing, such as parasites, diseases, food availability and quality, predators, and competitions, just to name a few. We also believe that small samples with a lack of replication in more than one population are likely to be the major cause for the large variability in RIs for the same species among different research groups [1]. From a methodological point of view, our results indicate that RIs obtained from a single population should be regarded with caution, even if computed over a large sample size, and reliable leukogram baselines should only be achieved by sampling a large number of populations from the whole species’ range.

The assumption that RIs are constant all over the range of the species did not find support in our analyses, as we detected a latitudinal cline of variation in leukocyte profile. From Southern to Northern populations, the WBC increases, the proportion of heterophils decreases while the opposite does occur in lymphocytes, leading the H:L ratio to decrease northwards. This pattern of variation is consistent with the ecology of the common wall lizard, which is a continental species of mainland Europe [55], and in Italy, it becomes increasingly rare from North to South, where it occurs only in mountains [56]. More in general, species that are spread over a large geographical area may experience different climatic conditions and may have consequently evolved local adaptations, which are reflected in the geographic pattern of variation of RIs in leukocyte differential count. Again, from a methodological point of view, the only way to account for such a pattern is to collect data from several populations, spread all over the species’ range.

Finally, we clearly showed that several components of the leukogram are characterized by a regular pattern of variation above and below the reference values during spring-summer, possibly reflecting seasonal variation in temperature or for other factors, namely hormone plasma levels [57, 58]. Indeed, reference values of WBC and lymphocytes sensibly decreased during the peak of the breeding season (May), whereas heterophils and eosinophils increased. Patterns of variation in leukogram over the breeding season appear to be a component common to several species of reptiles [33, 35], and our data indicate that seasonal oscillations around the baselines may exceed RIs. Therefore, if the seasonal pattern is ignored, some healthy individuals might be regarded as sick, leading to type I error (false positive). Baseline leukocyte profile also varies according to body size, which, in lizards, is a proxy for the age. In general, adults had more WBC, more heterophils, eosinophils, and monocytes, and fewer lymphocytes compared with juveniles and subadults. Thus, the leukocyte profile in this species seems to show senescence, as previously reported for other species [35]. Seasonal and age patterns we detected in common wall lizards are only two examples of how internal/external factors may affect leukocyte profile, but point out the need to account for such effects when trying to define leukogram’s RIs.

In conclusion, the application of WBC counts and leukocyte differential counts in conservation, as well as ecological research on reptiles, is still limited mainly because of the lack of basic information, but also by the inconsistencies among studies performed until now. These troubles mainly arise from the heterogeneity among methodological approaches and low standardization in experimental design. We argue that it is possible to disentangle local and geographic effects on leukocyte profile only by involving a large number of populations over a wide geographic range in *ad hoc* statistical models. Otherwise, the inconsistency among studies will carry on without a solution, and we may never get to leukocyte profiles reliable enough to be used to assess the health conditions of wild animals.
